# Patterns and Motivations for White Noise Use in Children: A Cross-Sectional Analysis of Parental Practices

**DOI:** 10.7759/cureus.114091

**Published:** 2026-08-06

**Authors:** Erin M Gawel, Alexandra F Corbin, Katelin R Keenehan, Gaayathri Varavenkataraman, Beatrice R Bacon, Michele M Carr

**Affiliations:** 1 Otolaryngology - Head and Neck Surgery, University at Buffalo Jacobs School of Medicine and Biomedical Sciences, Buffalo, USA

**Keywords:** children, hearing development, parental practices, pediatric otolaryngology, sleep aid, speech development, white noise

## Abstract

Introduction

White noise is commonly used as a pediatric sleep aid, but parental use patterns, motivations, and perceived developmental concerns remain poorly characterized. This study aimed to assess the frequency of white noise use among children presenting to a pediatric otolaryngology clinic, describe parental practices and motivations, and evaluate whether white noise use was associated with parental concerns about their child's speech or hearing development.

Methods

A cross-sectional survey was distributed to parents of children aged eight years or younger at a pediatric otolaryngology clinic affiliated with an academic tertiary care hospital from December 2023 to June 2024. Survey items assessed parental demographics, child age, reason for the clinic visit, concerns regarding speech and hearing development, and white noise use practices.

Results

A total of 253 parents completed the survey. White noise use was reported by 150 parents (59.3%). Among parents who used white noise, most used a white noise machine kept in the bedroom (n = 123, 82.0%) and played white noise overnight (n = 136, 90.7%). The most common motivations were improving sleep quality (n = 97, 64.7%) and masking environmental noise (n = 92, 61.3%). The mean daily exposure was 9.6 ± 2.6 hours. Parents who used white noise had younger children than those who did not (mean age: 3.3 vs 4.4 years; p < 0.001). White noise use was not associated with parental concerns about speech or hearing development.

Conclusions

White noise use appears to be common among parents of young children and is primarily used overnight to improve sleep quality and mask environmental noise. These findings support greater clinician awareness of pediatric white noise practices and highlight the need for evidence-based counseling on safe use, while acknowledging the limitations of parent-reported data, single-site sampling, and the absence of objective measurements of device sound output.

## Introduction

White noise is a continuous sound signal with equal intensity across the audible frequency range (20-20,000 Hz), producing a steady background noise that can mask environmental sounds. White noise machines and digital applications have become increasingly accessible and are commonly marketed to parents as tools to improve infant and child sleep [1,2].

White noise has been investigated for several potential benefits in pediatric populations, including promoting sleep, soothing colicky infants, reducing procedural pain, and improving attention or working memory in select children with attention-deficit/hyperactivity disorder [3-7]. However, the safety and long-term developmental effects of repeated white noise exposure in children remain incompletely studied in human subjects. Existing evidence raises concern that prolonged or high-intensity noise exposure may adversely affect auditory and neurodevelopmental outcomes, and some commercially available infant sleep devices may exceed recommended sound exposure thresholds when used at high volume or placed close to the child [8-10]. Despite widespread use, there is limited evidence-based guidance to help parents determine safe volume levels, device placement, and duration of exposure.

Given the increasing availability and use of white noise products, a better understanding of parental practices is needed to guide counseling in pediatric clinical settings. The primary objective of this study was to determine the frequency of white noise use among children presenting to a pediatric otolaryngology clinic. Secondary objectives were to characterize parental white noise use practices and motivations within this clinical population and to evaluate whether white noise use was associated with parent-reported concerns regarding their children's speech or hearing development.

## Materials and methods

Study design and participants

This cross-sectional survey study was conducted at a pediatric otolaryngology clinic affiliated with an academic tertiary care hospital from December 2023 to June 2024. The study protocol was approved by the Institutional Review Board at the University at Buffalo. Verbal consent was obtained from participants at the time of recruitment. Eligible participants were parents or guardians of children aged eight years or younger presenting to the clinic during the study period. Because the study was designed as a clinic-based cross-sectional survey, eligible participants were recruited consecutively as they presented for scheduled appointments, resulting in a convenience sample. This approach was chosen to efficiently capture parental white noise practices among families seeking pediatric otolaryngologic care during the study period. Because the study was conducted at a single specialty clinic, the sample may not be fully representative of the broader population of parents and guardians of young children. No organization was paid or contracted to assist with participant recruitment, data collection, data management, or analysis. No artificial intelligence tools were used to conduct the study or analyze the data.

Survey instrument and data collection

A paper survey developed by the study authors was distributed to eligible parents or guardians. The full survey questionnaire is provided in the Appendix. The primary outcome was parent-reported white noise use for the child. Parents were classified as white noise users if they answered "yes" to the survey question, "Have you ever used white noise for your child?", while parents who answered "no" were classified as non-users. Secondary outcomes included white noise device type, timing of use, daily duration of exposure, reasons for use, sources of white noise recommendations, and parental concerns about the child’s hearing or speech development. The survey collected parent demographic information, including sex, age, race, ethnicity, highest level of education, and whether the parent worked in the medical field. Race and ethnicity were self-reported by participants and were collected to describe the study sample.

These variables were treated as social, not biological, constructs. Child-related variables included age, reason for the clinic visit, parental concerns about hearing, and parental concerns about speech development. Hearing and speech development concerns were each assessed using a four-point ordinal scale: not at all, a little, quite a bit, and very much. Parents who reported using white noise for their child completed additional questions about white noise practices, including the type of device or output used, the time of day white noise was used, the estimated number of hours per day the child was exposed to white noise during the past week, reasons for using white noise, and how the parent learned about white noise use.

Statistical analysis

Statistical analyses were performed using SPSS Statistics version 29.0 (IBM Corp., Armonk, NY). Continuous variables were summarized as means ± standard deviations (SD), and categorical variables were summarized as frequencies and percentages. Comparisons between parents who did and did not report white noise use were performed using Student's t tests for continuous variables and chi-square tests or Fisher's exact tests for categorical variables, with Fisher's exact test used when more than 20% of cells in a contingency table had expected frequencies less than 5. Variables associated with white noise use in bivariate analyses, as well as prespecified demographic characteristics, were further evaluated using multivariable binary logistic regression. To address sparse categorical data, race was recoded as White and non-White categories for multivariable analyses. Adjusted odds ratios (OR) with 95% confidence intervals (CI) were reported. All analyses were prespecified, and statistical significance was defined as a two-sided p-value less than 0.05.

## Results

Parent demographics

A total of 253 parents completed the survey, including 150 (59.3%) who reported using white noise for their child and 103 (40.7%) who did not. Most respondents were female (n = 204, 80.6%), had a mean age of 35.8 ± 5.5 years, and identified as White (n = 230, 90.9%). Parent age, sex, ethnicity, and employment in the medical field did not differ significantly between parents who reported white noise use and those who did not. Race differed significantly between groups (p = 0.004), with a greater proportion of parents reporting white noise use identifying as White (143/150, 95.3%). White noise use also varied by educational attainment (p = 0.003), with high school graduates representing a greater proportion of non-users (35/103, 34.0%) than users (21/150, 14.0%), whereas parents with a college or postgraduate/professional education represented a greater proportion of white noise users (Table 1).

**Table 1:** Parent demographic characteristics stratified by white noise use P-values were calculated using Student's t-test^a^, chi-square test^b^, or Fisher's exact test^c ^as appropriate SD: standard deviation

Characteristic	Total (n = 253)	White noise use (n = 150)	No white noise use (n = 103)	P-value
Age, years, mean ± SD	35.8 ± 5.5	35.3 ± 4.7	36.7 ± 6.6	0.055^a^
Sex, n (%)				0.437^b^
Male	48 (19.0)	26 (17.3)	22 (21.4)	
Female	204 (80.6)	123 (82.0)	81 (78.6)	
Race, n (%)				0.004^c^
Asian	8 (3.2)	2 (1.3)	6 (5.8)	
Black/African American	7 (2.8)	4 (2.7)	3 (2.9)	
White	230 (90.9)	143 (95.3)	87 (84.5)	
Mixed	5 (2.0)	0 (0.0)	5 (4.9)	
Ethnicity, n (%)				0.688^c^
Hispanic/Latino	6 (2.4)	3 (2.0)	3 (2.9)	
Not Hispanic/Latino	239 (94.5)	143 (95.3)	96 (93.2)	
Employment in medical field, n (%)				0.535^b^
Yes	66 (26.1)	37 (24.7)	29 (28.2)	
No	187 (73.9)	113 (75.3)	74 (71.8)	
Education, n (%)				0.003^c^
Some high school	4 (1.6)	3 (2.0)	1 (1.0)	
High school graduate	56 (22.1)	21 (14.0)	35 (34.0)	
College graduate	102 (40.3)	66 (44.0)	36 (35.0)	
Postgraduate/professional	88 (34.8)	57 (38.0)	31 (30.1)	

Child visit characteristics

The mean age of children included in the study was 3.8 ± 2.3 years. Children whose parents reported white noise use were significantly younger than those whose parents did not report white noise use (3.3 ± 2.2 vs. 4.4 ± 2.3 years, p < 0.001). Most parents reported no concerns about their child’s hearing (n = 178, 70.4%) or speech development (n = 162, 64.0%), and parent-reported concerns regarding both hearing and speech development did not differ significantly between groups. The most common reasons for the child’s clinic visit were follow-up appointment (n = 63, 24.9%), tympanostomy tube check (n = 49, 19.4%), and recurrent ear infections (n = 43, 17.0%). Tympanostomy tube check was the only visit reason significantly associated with white noise use (p = 0.010), with a greater proportion of children in the white noise use group presenting for tympanostomy tube evaluation (37/150, 24.7%) compared with the no white noise use group (12/103, 11.7%; Table 2).

**Table 2:** Child visit characteristics and parent-reported hearing and speech concerns stratified by white noise use P-values were calculated using Student's t-test^a^, Fisher's exact test^b^, or chi-square test^c^ as appropriate SD: standard deviation

Characteristic/concern	Total (n = 253)	White noise use (n = 150)	No white noise use (n = 103)	P-value
Child age, years, mean ± SD	3.8 ± 2.3	3.3 ± 2.2	4.4 ± 2.3	< 0.001^a^
Have you had concerns about your child’s hearing? N (%)				0.189^b^
Not at all	178 (70.4)	111 (74.0)	66 (64.1)	
A little	62 (24.5)	33 (22.0)	29 (28.2)	
Quite a bit	11 (4.3)	6 (4.0)	5 (4.9)	
Very much	2 (0.8)	0 (0.0)	2 (1.9)	
Have you had concerns about your child’s speech development? N (%)				0.964^c^
Not at all	162 (64.0)	95 (63.3)	66 (64.1)	
A little	51 (20.2)	31 (20.7)	20 (19.4)	
Quite a bit	22 (8.7)	12 (8.0)	10 (9.7)	
Very much	18 (7.1)	11 (7.3)	7 (6.8)	
Reason for visit, n (%)				
Follow-up	63 (24.9)	42 (28.0)	21 (20.4)	0.234^c^
Tympanostomy tube check	49 (19.4)	37 (24.7)	12 (11.7)	0.010^c^
Recurrent ear infections	43 (17.0)	22 (14.7)	21 (20.4)	0.664^c^
Postoperative appointment	34 (13.4)	19 (12.7)	15 (14.6)	0.435^c^
Unsure/referred from primary physician	33 (13.0)	18 (12.0)	15 (14.6)	0.409^c^
Enlarged tonsils/adenoids	32 (12.6)	21 (14.0)	11 (10.7)	0.169^c^
Sleep concerns/snoring	27 (10.7)	18 (12.0)	9 (8.7)	0.191^c^
Chronic nasal congestion/cough	17 (6.7)	7 (4.7)	10 (9.7)	0.131^b^
Hearing concerns/speech delay	16 (6.3)	7 (4.7)	9 (8.7)	0.116^c^
Ear drainage/ear wax	10 (4.0)	4 (2.7)	6 (5.8)	0.325^b^
Recurrent strep infections	9 (3.6)	6 (4.0)	3 (2.9)	0.742^b^
Breathing issues/stridor	8 (3.2)	5 (3.3)	3 (2.9)	1^b^
Hemangioma	7 (2.8)	5 (3.3)	2 (1.9)	0.552^c^
Nosebleeds	4 (1.6)	3 (2.0)	1 (1.0)	0.648^b^
Tongue tie	2 (0.8)	0 (0.0)	2 (1.9)	0.165^b^
Neck abscess	1 (0.4)	0 (0.0)	1 (1.0)	1^b^

Factors independently associated with white noise use

Multivariable binary logistic regression identified younger child age as independently associated with white noise use (OR: 0.84, 95% CI: 0.73-0.96; p = 0.011). Race also remained significantly associated with white noise use, with non-White participants having lower odds of reporting white noise use than White participants (OR: 0.25, 95% CI: 0.08-0.81; p = 0.021). Tympanostomy tube check was no longer significantly associated with white noise use after adjustment (p = 0.416). Parent age, sex, ethnicity, employment in the medical field, and education were not significantly associated with white noise use after adjustment (Table 3).

**Table 3:** Multivariable logistic regression of factors associated with white noise use OR: odds ratio; CI: confidence interval

Variable	Adjusted OR (95% CI)	P-value
Child age, years	0.84 (0.73–0.96)	0.011
Parent age, years	0.97 (0.91–1.03)	0.261
Sex		
Male (reference)	—	—
Female	1.18 (0.57–2.46)	0.654
Ethnicity		
Non-Hispanic/Latino (reference)	—	—
Hispanic/Latino	0.85 (0.14–5.28)	0.863
Employment in medical field		
Not employed (reference)	—	—
Employed	0.68 (0.36–1.31)	0.250
Education		
Some high school (reference)	—	—
High school graduate	0.20 (0.02–2.22)	0.190
College graduate	0.60 (0.06–6.60)	0.677
Postgraduate/professional	0.58 (0.05–6.59)	0.662
Race		
White (reference)	—	—
Non-White	0.25 (0.08–0.81)	0.021
Tympanostomy tube check		
No (reference)	—	—
Yes	1.37 (0.64–2.96)	0.416

White noise use practices

White noise use was reported by more than half of parents (n = 150, 59.3%). Among the 150 parents who used white noise, most used a white noise machine kept in the bedroom (n = 123, 82.0%) and used white noise overnight (n = 136, 90.7%), with a mean duration of 9.6 hours per day (95% CI: 9.2-10.1). The most common reasons for use were improving sleep quality (n = 97, 64.7%) and masking environmental noise (n = 92, 61.3%). Friends and family were the most common sources of white noise recommendations (n = 87, 58.0%). Additional white noise use practices are presented in Table 4.

**Table 4:** White noise use practices among surveyed parents Percentages for questions permitting multiple responses were calculated among parents who reported white noise use; therefore, percentages may total more than 100% SD: standard deviation

Survey question	Value
Do you use white noise for your child?	N (%)
Yes	150 (59.3)
No	103 (40.7)
What type of device/output do you use to play white noise? Select all that apply	
White noise machine kept in bedroom	123 (82.0)
Mobile application	11 (7.3)
Fan	11 (7.3)
Online video	10 (6.7)
Portable white noise machine kept on the car seat and/or stroller	5 (3.3)
At what time do you play white noise for your child? Select all that apply	
Morning	7 (4.7)
Afternoon	26 (17.3)
Evening	33 (22.0)
Overnight	136 (90.7)
Why do you play white noise for your child? Select all that apply	
Improve sleep quality	97 (64.7)
Cover environmental noise	92 (61.3)
Get to sleep more quickly	72 (48.0)
Soothe back to sleep when child wakes	61 (40.7)
Reduce stress for the child	32 (21.3)
Reduce crying	10 (6.7)
How did you learn about using white noise for your child? Select all that apply	
Friend/family member	87 (58.0)
Social media (Facebook, Twitter, TikTok, Instagram)	27 (18.0)
Healthcare provider (physician, physician assistant, nurse)	24 (16.0)
Search engine (Google, Bing, Safari)	15 (10.0)
News or magazine article	4 (2.7)
Personal experience	4 (2.7)
Newborn parenting books	2 (1.3)
Scholarly articles	1 (0.7)
In the last week, how many hours per day was your child exposed to white noise? mean ± SD	9.6 ± 2.6

## Discussion

White noise was commonly used in our sample, primarily as part of children’s sleep routines to improve sleep and reduce disruptive background noise. In adjusted analyses, younger child age and race were associated with reported white noise use, whereas parent age, sex, ethnicity, employment in the medical field, education, and presentation for a tympanostomy tube check were not significantly associated with use. White noise use was also not significantly associated with parent-reported concerns about hearing or speech development. These findings characterize patterns of reported white noise use but should not be interpreted as evidence that white noise causes or prevents hearing or speech concerns.

The association between younger child age and white noise use may reflect greater use of white noise as a sleep and soothing aid during infancy and early childhood, which has been the focus of much of the existing pediatric white noise literature [7,9,11,12]. The observed association with race warrants cautious interpretation. Previous research has identified racial and ethnic differences in preschool bedtime routines, sleep timing, and other sleep-related practices, which may be shaped by social, cultural, economic, and environmental circumstances [13,14]. However, the present study did not directly evaluate these contextual factors. In addition, the relatively small number of non-White participants required race to be combined into White and non-White categories for the multivariable analysis, limiting the ability to examine differences among individual racial groups. Therefore, the observed association should be considered exploratory, and further investigation in more diverse populations is needed to better understand demographic differences in white noise use.

Previous studies have supported the potential benefits of white noise for sleep and soothing in children. One randomized controlled trial found that 80% of neonates exposed to white noise fell asleep spontaneously within five minutes, compared with 25% of neonates in the control group [7]. Another study found that white noise improved sleep among premature infants, significantly increasing both sleep duration and sleep efficiency [11]. Combining white noise with a recording of the mother’s heartbeat has also been associated with improvements in sleep and developmental progression among premature infants in the neonatal intensive care unit [12]. White noise may additionally have a calming effect on infants with colic, with one study reporting reduced crying duration and increased sleep duration compared with swinging, a more traditional soothing method [6]. Together, these findings provide context for why parents may incorporate white noise into children’s sleep routines.

In addition to its potential sleep-related benefits, white noise has been studied as a nonpharmacologic method for reducing pain in newborns and premature infants. Prior studies have reported reduced pain during procedures such as vaccinations, blood draws, and endotracheal suctioning using measures such as the Neonatal Infant Pain Scale and Premature Infant Pain Profile [15-19]. For example, premature infants exposed to white noise during vaccination demonstrated lower pain scores than infants in a control group [16]. Similarly, newborns exposed to white noise during invasive procedures reportedly cried for shorter durations than those not exposed to white noise [15]. White noise may therefore serve as an adjunctive tool for infant comfort, either alone or in combination with other soothing methods such as holding, maternal voice recordings, or oral glucose solution [20,21].

White noise has also been evaluated in children with attention-deficit/hyperactivity disorder and reading difficulties. Studies have associated white noise exposure with improved working memory, enhanced word recall, and reduced off-task behavior in select children [3,22,23]. Given that attention-deficit/hyperactivity disorder frequently overlaps with reading disabilities, findings suggesting that white noise may improve reading skills and memory recall among children with reading disabilities are notable [24]. However, these effects may vary according to baseline attentional ability, and white noise may not provide the same benefits for typically developing children or children with normal attentiveness [10]. Together, these studies suggest that white noise may have potential cognitive or behavioral benefits in specific pediatric populations. However, such benefits should not be inferred from the present data because attention, learning, and neurocognitive outcomes were not evaluated.

Despite its possible benefits, prolonged or high-volume white noise exposure may carry risks. At maximum volume, some white noise devices have been shown to exceed recommended noise exposure levels, raising concern for noise-induced hearing loss, particularly when devices are placed close to infants or used for extended periods [25,26]. Because our study relied on parent-reported survey data, objective measures of device sound output, volume settings, and distance from the child were not collected. These variables cannot be accurately determined through parent report and would require direct acoustic measurement. Although further research is needed to define optimal sound levels and exposure durations in children, a recent scoping review suggested limiting continuous white noise exposure during sleep to no more than 16 hours per day [9]. Evidence from other populations also suggests that the effects of white noise may vary by intensity. Among neurotypical young adults, exposure to 45-decibel white noise was associated with improved cognitive performance, creativity, and stress levels, whereas exposure to 65-decibel white noise was associated with increased stress [27]. These findings emphasize the importance of considering volume, distance, and duration when counseling families about white noise use [28].

The absence of a significant association between white noise use and parental concerns about speech or hearing development should be interpreted cautiously. This finding may indicate that parents do not perceive white noise as a potential risk to speech or hearing, particularly because many participants reported learning about white noise from friends, family members, or social media. Alternatively, typical household white noise use may not be associated with noticeable speech or hearing concerns. However, this study relied on parent-reported concerns rather than formal audiologic testing or speech-language evaluation. Further investigation is therefore needed to determine whether white noise exposure is associated with objective developmental outcomes. Animal studies have shown that prolonged noise exposure may disrupt auditory cortical development and neuropsychological outcomes, but the applicability of these findings to children exposed to household white noise remains unclear [8,29].

Given the prominent role of informal recommendation sources in this study, healthcare providers should consider discussing white noise use with parents, particularly in pediatric otolaryngology settings, to help families make informed decisions. Clinicians should acknowledge that some evidence suggests white noise may be helpful for sleep routines in certain children while also advising families to use the lowest effective volume, place devices away from the child, and avoid excessive exposure durations [25,26,28]. Importantly, despite the widespread use of white noise observed among young children in this study, insufficient evidence is available to define safe sound intensity, device placement, and exposure duration. Future studies should evaluate these exposure parameters in relation to hearing, speech, sleep, and neurodevelopmental outcomes to establish evidence-based clinical guidelines.

Limitations and future research directions

This study has several limitations. First, it was conducted at a single pediatric otolaryngology clinic, which may limit its generalizability to broader pediatric populations. Second, the sample was relatively small and predominantly composed of White female respondents, which may limit interpretation across more diverse parent populations. Race and ethnicity were self-reported and should be interpreted as social rather than biological variables. Third, white noise use, daily duration, motivations, and developmental concerns were parent-reported, which may have introduced recall or reporting bias. Another key limitation was the lack of data regarding device volume, device placement, and sound output levels, which prevented assessment of potential risks related to noise intensity.

In addition, children whose parents reported white noise use were significantly younger than those whose parents did not, and the study population included children presenting with a heterogeneous range of otolaryngologic conditions. Although child age and presentation for a tympanostomy tube check were included in the adjusted analysis, residual confounding from measured or unmeasured factors remains possible. Hearing and speech outcomes were also based solely on parent-reported concerns rather than objective audiologic or speech-language assessments. Furthermore, the survey instrument was developed specifically for this study and was not formally validated. Because the numbers of eligible participants approached and those who declined participation were not systematically recorded, a response rate could not be calculated. Finally, because of the cross-sectional design, this study cannot establish causal relationships between white noise use and parent-reported concerns regarding hearing or speech development.

Future research is needed to better understand appropriate sound levels, exposure durations, and the potential effects of white noise on hearing, speech, sleep, and neurodevelopment in pediatric populations. Follow-up studies should quantify sound exposure from specific white noise devices and evaluate these outcomes using objective audiologic, speech-language, sleep, and developmental measures.

## Conclusions

White noise use is common among young children and is most often used during sleep to improve sleep quality and mask environmental noise. Although existing evidence suggests that white noise may be beneficial for some families, its long-term effects on child development remain unclear, particularly with prolonged or high-volume exposure. Given that many parents learn about white noise from informal sources such as friends, family, and social media, pediatric healthcare providers should consider routinely asking about white noise use and providing practical guidance on safe listening practices.

## Figures and Tables

**Figure 1:**
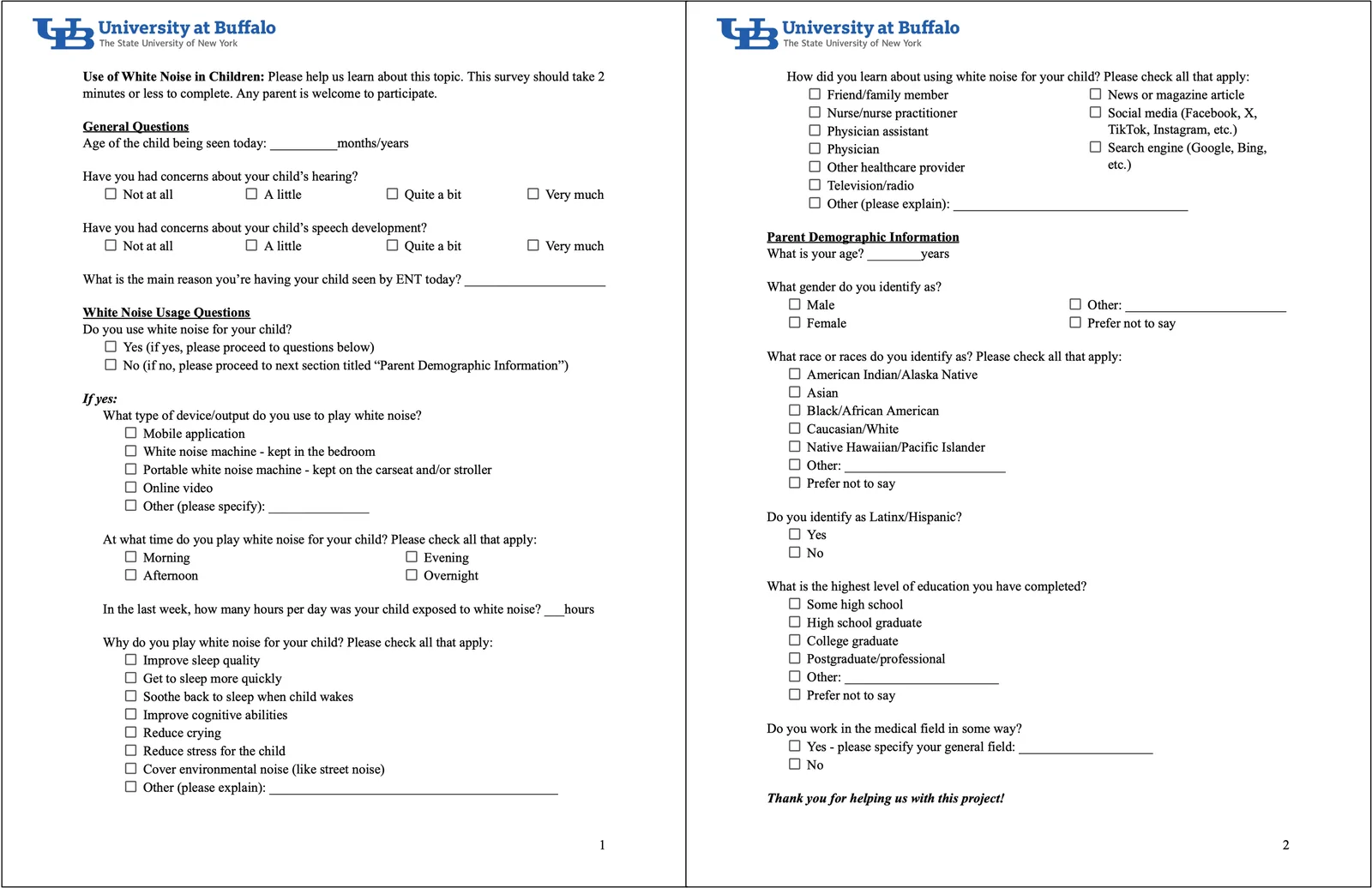
Survey questionnaire
